# Association between Perceived Neighborhood Built Environment and Walking and Cycling for Transport among Inhabitants from Latin America: The ELANS Study

**DOI:** 10.3390/ijerph17186858

**Published:** 2020-09-19

**Authors:** Gerson Ferrari, André Oliveira Werneck, Danilo Rodrigues da Silva, Irina Kovalskys, Georgina Gómez, Attilio Rigotti, Lilia Yadira Cortés Sanabria, Martha Cecilia Yépez García, Rossina G. Pareja, Marianella Herrera-Cuenca, Ioná Zalcman Zimberg, Viviana Guajardo, Michael Pratt, Cristian Cofre Bolados, Emilio Jofré Saldía, Carlos Pires, Adilson Marques, Miguel Peralta, Eduardo Rossato de Victo, Mauro Fisberg, on behalf of the ELANS Study Group

**Affiliations:** 1 Laboratorio de Ciencias de la Actividad Física, el Deporte y la Salud, Facultad de Ciencias Médicas, Universidad de Santiago de Chile, USACH, Santiago 7500618, Chile; cristian.cofre@usach.cl (C.C.B.); emilio.jofre.s@usach.cl (E.J.S.); 2 Department of Nutrition, School of Public Health, Universidade de São Paulo (USP), São Paulo 01246-904, Brazil; andreowerneck@gmail.com; 3 Department of Physical Education, Federal University of Sergipe—UFS, São Cristóvão 49100-000, Brazil; danilorpsilva@gmail.com; 4 Carrera de Nutriciόn, Facultad de Ciencias Médicas, Pontificia Universidad Catόlica Argentina, Buenos Aires C1107 AAZ, Argentina; ikovalskys@gmail.com; 5 Departamento de Bioquímica, Escuela de Medicina, Universidad de Costa Rica, San José 11501-2060, Costa Rica; georgina.gomez@ucr.ac.cr; 6 Centro de Nutrición Molecular y Enfermedades Crónicas, Departamento de Nutrición, Diabetes y Metabolismo, Escuela de Medicina, Pontificia Universidad Católica, Santiago 8330024, Chile; arigotti@med.puc.cl; 7 Departamento de Nutrición y Bioquímica, Pontificia Universidad Javeriana, Bogotá 110231, Colombia; ycortes@javeriana.edu.co; 8 Colégio de Ciencias de la Salud, Universidad San Francisco de Quito, Quito 17-1200-841, Ecuador; myepez@usfq.edu.ec; 9 Instituto de Investigación Nutricional, Lima 15026, Peru; rpareja@iin.sld.pe; 10 Centro de Estudios del Desarrollo, Universidad Central de Venezuela (CENDES-UCV)/Fundación Bengoa, Caracas 1053, Venezuela; manyma@gmail.com; 11 Departamento de Psicobiologia, Universidade Federal de São Paulo, São Paulo 04023-062, Brazil; iona.zimberg@gmail.com; 12 Nutrition, Health and Wellbeing Area, International Life Science Institute (ILSI) Argentina, Santa Fe Av. 1145, CABA C1059ABF, Argentina; viviana.guajardo@comunidad.ub.edu.ar; 13 Institute for Public Health, University of California San Diego, La Jolla, CA 92093-0021, USA; mipratt@health.ucsd.edu; 14 Facultad de Salud, Instituto de Ciencias del Deporte, Escuela de Ciencias del Deporte y la Actividad Fìsica, Universidad Santo Tomas Santiago de Chile, Santiago 5520540, Chile; 15 Center for Research in Neuropsychology and Cognitive and Behavioral Intervention (CINEICC), Faculty of Psychology and Educational Sciences, University of Coimbra, 3000-115 Coimbra, Portugal; carlosandrepires@gmail.com; 16 CIPER, Faculdade de Motricidade Humana, Universidade de Lisboa, 1499-002 Lisbon, Portugal; adncmpt@gmail.com (A.M.); miguel.peralta14@gmail.com (M.P.); 17 ISAMB, Faculdade de Medicina, Universidade de Lisboa, 1649-028 Lisbon, Portugal; 18 Departamento de Pediatria da Universidade Federal de São Paulo, São Paulo 04023-061, Brazil; eduardorossato93@gmail.com (E.R.d.V.); mauro.fisberg@gmail.com (M.F.); 19 Instituto Pensi, Fundação José Luiz Egydio Setubal, Hospital Infantil Sabará, São Paulo 01227-200, Brazil

**Keywords:** transport physical activity, walking, cycling, neighborhood built environment, Latin America

## Abstract

Purpose: This study aimed to examine the associations of the perceived neighborhood built environment with walking and cycling for transport in inhabitants from Latin American countries. Methods: This cross-sectional study involved 9218 participants (15–65 years) from the Latin American Study of Nutrition and Health, which included a nationally representative sample of eight countries. All participants completed the International Physical Activity Questionnaire-Long Form for measure walking and cycling for transport and the Neighborhood Environment Walkability Scale-Abbreviated. Furthermore, perceived proximity from home to public open spaces and shopping centers was assessed. Results: Perceived land use mix-access (OR: 1.32; 95%CI: 1.16,1.50) and the existence of many alternative routes in the neighbourhood (1.09 1.01,1.17) were associated with higher odds of reporting any walking for transport (≥10 min/week). Perceived slow speed of traffic (1.88 1.82,1.93) and few drivers exceeding the speed limits (1.92; 1.86,1.98) were also related to higher odds of reporting any walking for transport. The odds of reporting any cycling for transport (≥10 min/week) were higher in participants perceiving more walking/cycling facilities (1.87 1.76,1.99), and better aesthetics (1.22 1.09,1.38). Conclusions: Dissimilar perceived neighborhood built environment characteristics were associated with walking and cycling for transport among inhabitants from Latin America.

## 1. Introduction

Physical activity has benefical effects on numerous health outcomes. Higher physical activity levels decrease the risk of cardiovascular disease (CVD), including coronary heart disease, type 2 diabetes, colon cancer, and breast cancer, as well as increasing life expectancy [1]. Active transportation (i.e., walking and cycling for travel purposes) has been recommended as a practical way of incorporating more physical activity into daily life [2] and those who engage in active transportation tend to be more active in duration and frequency than those without active transportation [3]. Furthermore, a systematic review and meta-analysis of 531,333 participants reported that active transportation had a significantly reduced risk of all-cause mortality, cardiovascular disease incidence and diabetes [4].

Recently, Latin America has undergone a rapid urbanization process with demographic, epidemiological and socioeconomic changes and is currently the most urbanized region in the world (around 80% of Latin Americans live in cities) [5]. The urbanization change people’s behaviour and in most cases decrease the physical activity levels. Between 2001 and 2016, the prevalence of physical inactivity (not meeting the physical activity recommendation proposed by the World Health Organization) increased by more than 5 percentage points in Latin America (from 33.4% in 2001 to 39.1% in 2016) [6]. Overall, the prevalence of physical inactivity (<150 min/week in moderate-to-vigorous physical activity) ranging from 26.9% (Chile) to 47% (Costa Rica and Venezuela). Physical inactivity (>40%) was prevalent in Brazil (43.5%), Argentina (43.7%), Venezuela (47.1%), and Costa Rica (48.0%) [7]. Furthermore, the highest mean of total sedentary behavior per day were in Costa Rica (524.6 min/day) followed by Brazil (455.1 min/day) [8]. The Latin American region is characterized by high population density, disorganized and heavy traffic, air and noise pollution, rising crime rates, high-income inequality, high levels of poverty, and population aging [9,10]. The dissimilar features of Latin American countries reduce, to translate findings from high-income countries (e.g., the United States, European countries) to this particular region. Therefore, the investigation of how the built environment can influence physical activity during transportation is warranted.

Different studies have explored associations between neighborhood built environmental (destination accessibility, street connectivity, recreational facilities, and public transport) and walking or cycling for transport [11,12,13] and found that characteristics related to active transport infrastructure, connectivity, walkability, safety and aesthetics were associated with higher physical activity during transportation. Nonetheless, most previous research was done in the high-income countries [14,15,16]. Only one of the existing Latin American studies used a representative sample of the urban population which may limit the generalizability of the observed associations, but it was related to a representative sample of only one city per country [13,17,18] and had not sufficient power and variability to assess walking and cycling separately. Therefore, the purpose of the present study was to examine the associations of the perceived neighborhood built environment with walking and cycling for transport in a representative sample of inhabitants from eight Latin America countries.

## 2. Material and Methods

### 2.1. Study Design and Sample

We use data from Estudio Latinoamericano de Nutrición y Salud (Latin American Study of Nutrition and Health, ELANS—https://es.elansstudy.com/). ELANS was an observational, cross-sectional, epidemiological multi-country (Argentina, Brazil, Chile, Colombia, Costa Rica, Ecuador, Peru, and Venezuela) study that uses a common design and comparable methods across countries. ELANS analyses a large representative sample from these eight countries and focuses on urban populations [19]. The main criteria for countries participate in ELANS were as follows: (a) data were collected from participants (15–65 years of age) selected; (b) at least the minimum number of participants per country according to the sample size; c) investigators were selected for the ELANS study based on their ability to collect the data which included investigator experience, funding availability, completed pilot work and having translated ELANS materials and diversity of their geographic location in Latin America.

Data collection dates ranged from September 2014 to February 2015. The overarching ELANS protocol was approved by the Western Institutional Review Board (#20140605) and is registered at ClinicalTrials.gov (#NCT02226627). Ethical approval was obtained from each local institutional review board (Brazil: 689.294; Chile: 14-179; Costa Rica: VI-6480-2013; Ecuador: 2014-057M; Peru: 352-2014/CIEI-IIN), and participants’ informed consent was obtained before data collection.

The entire ELANS study consisted of 9218 (4809 women; aged 15–65 years) participants who were chosen using a random complex, multistage sample design, stratified by conglomerates, with all regions of each country represented, and random selection of Primary Sampling Units (PSUs) and Secondary Sampling Units (SSUs) according to probability proportional to size method. The PSUs were areas (e.g., counties, municipalities, neighborhoods, residential areas) within each selected city in each country. An “n” size proportional to the population weight was used for the range of PSUs. In this instance, a simple random sampling of “n” with replacement was achieved to adhere to the principle of statistical independence of the selection of the areas included in the PSU sample. For these random selections, the probability proportional to size (PPS) method was applied. Therefore, within each of the areas included in the PSU allocation, a representative sample of SSUs was randomly selected using the PPS method.

For the selection of households, we implemented a four-step, systematic randomization procedure by establishing a selection interval (k): a) the total urban population was used to proportionally define the main regions and to select cities representing each region, including key cities and other representative cities in the region, using a random method and sampling criteria, while attempting to cover the determined urban population; b) the sampling points (survey tracts) of each city were randomly designated, and c) clusters of households were selected from each sampling unit. Addresses were chosen systematically using standard random route techniques, beginning with an initial address designated at random. The households were designated with three regular jumps; that is, a particular household was selected by randomly picking the first home and subsequently skipping three households; d) the designated respondent within each household was selected using the birthday method (half chosen using the next birthday; the other half using the last birthday).

In total, a number of 92 cities were participants in the study and the sampling size essential for sufficient accuracy was calculated with a 95% confidence level and a maximum error of 3.5% and a survey design effect of 1.75 based on guidance from the National Center for Health Statistics [20], and calculations of the minimum sample sizes required per sex, age group and socioeconomic status were performed for each country [19,21]. The exclusion criteria adopted were: (a) pregnant and lactating women; (b) persons with physical or mental disabilities; (c) unsigned consent form; (d) individuals living in non-family residential environments; and (e) individuals who could not read. More information on the ELANS study is provided in Fisberg et al. [19].

The perceived neighborhood built environment and walking and cycling for transport procedure used in ELANS consist of self-reported data collected by questionnaires. The questionnaires (perceived neighborhood built environment and walking and cycling for transport) used in the ELANS were interviewer-administered during the home visit, and 9218 (15–65 years old) adults had complete data.

### 2.2. Perceived Neighborhood Built Environment

The Neighborhood Environment Walkability Scale-Abbreviated (NEWS-A) adapted for the current study was used to collect data of perceived neighborhood built environment [22]. The NEWS-A was translated from English into the language of the participanting countries (Spanish and Portuguese) and the scale adaptation also encompassed the addition of two items to the safety from crime subscale, an item measuring the proximity of shopping centers and three items gauging the proximity to three types of public open space (metropolitan parks, playgrounds and public squares). The reliability and validity survey of NEWS-A have been previously shown in several countries with all included scales having test-retest reliability intraclass correlations >0.50 [23,24].

The NEWS-A is one of several recently developed questionnaires designed to measure residents’ perceptions of the environmental attributes of their local area [25]. The NEWS was designed to obtain residents’ perceptions of how neighborhood characteristics found in the transportation and urban planning literature were related to a higher frequency of walking and cycling for transport [26].

The NEWS-A include items that represent seven subscales: land use mix–diversity, land use mix-access, street connectivity, walking/cycling facilities, aesthetics, safety from traffic, and safety from crime (Table 1) [22]. The land use mix-diversity scale is assessed by the perceived walking proximity from home to twenty-three different types of destinations, with responses ranging from 1–5-min walking distance (coded as 5 = high walkability) to >30-min walking distance (coded as 1 = low walkability). The remaining six scales are average ratings of items answered on a four-point Likert scale (1 = strongly disagree to 4 = strongly agree). Scales were scored in a direction consistent with higher walkability and safety, with individual items reversed when necessary. Scoring details are described elsewhere [22]. Cronbach’s alpha was used to measure the internal consistency of the neighborhood environment characteristics’ scales. The scales ‘street connectivity’ (Cronbach’s alpha: 0.433) and ‘safety from traffic’ (Cronbach’s alpha: 0.191) from NEWS-A were not included in the results due to low internal consistency. Instead, the individual items were analysed separately. On the other hand, the Cronbach’s alpha was higher for ‘land use mix-diversity´ (Cronbach´s alpha: 0.897), ‘land use mix-access’ (Cronbach´s alpha: 0.693), ‘walking/cycling facilities’ (Cronbach´s alpha: 0.613), ‘aesthetics’ (Cronbach´s alpha: 0.804), ‘safety from crime’ (Cronbach´s alpha: 0.805), ‘distance to parks’ (Cronbach´s alpha: 0.620).

### 2.3. Walking and Cycling for Transport

Participants reported their walking and cycling for tranport levels by completing the long-form of the last seven days, interview version of the International Physical Activity Questionnaire (IPAQ) in Spanish and Portuguese [27]. We used only the questions that covered the active transport-related domain [21]. The IPAQ has been validated to assess PA in individuals aged 15–69 years in several countries [28,29].

The participants were instructed to report the frequency and duration (bouts of ≥10 min) of walking and cycling for transport domain. Specifically, the following questions were asked: (a) “During the last seven days, did you walk or use a bicycle (pedal cycle) for at least 10 min continuously to get to and from places?” (Yes, No); (b) “During the last seven days, on how many days did you walk or ride a bicycle for at least 10 min at a time to go from place to place?”; (c) “How much time did you usually spend on one of those days to bicycle or walk from place to place?” These questions were asked separately for walking and cycling. IPAQ walking and cycling for transport data are reported as min/week. Time (min/week) spent in each activity (i.e., walking and cycling) was calculated and used in the analysis. In this study, we used walking and cycling for transport separately. Details on the assessment of walking and cycling for transport by IPAQ have been published elsewhere [21].

### 2.4. Sociodemographic Characteristics

Information about demographics including age by year (15–65 years), and sex was collected using standard questionnaires. Socioeconomic status was evaluated by questionnaire using country-specific definitions based on national norms, laws, and the questionnaires used on national surveys in each country and included equivalent characteristics for all countries. Given the variablility in categoririzing socioeconomic status, a standard three level system (low, medium, high) was developed and included equivalent characteristics for all countries [30,31,32,33,34,35,36]. Detailed information can be found in a previous publication [19].

### 2.5. Statistical Analysis

Data analyses were performed with IBM SPSS, v.22 (SPSS Inc., IBM Corp., Armonk, New York, NY, USA). Descriptive statistics included mean, standard deviations (SD), and percentages were presented. Weighting was done considering sociodemographic characteristics, sex, socioeconomic level, and country [19].

Multilevel logistic regression models, with the individual as the first level and the country as the second level, were used to estimate the overall associations of neighborhood characteristics with walking and cycling for transport (odds ratio: OR; confidence interval 95%: 95%CI) with a binary dependent variable (0 = “<10 min of walking or cycling/week”, 1 = “≥10 min of walking or cycling/week”). Multilevel logistic regression modelling is a statistical method used to estimate the odds that an event will occur (yes/no outcome) while taking the dependency of data into account—participants from the same country are more likely to function in the same way than participants from different countries. This valuable information is taken into account when estimating the effects of independent variables on the outcome [37].

We present the results of walking and cycling for transport separately. Models were adjusted for sex, age, and socioeconomic level—these variables were included as independent variables in the logistic regression. We present the overall and country-specific results. A significance level of 5% was considered (*p* < 0.05).

## 3. Results

### 3.1. Descriptive Results

Table 2 describes the participants’ characteristics. The total sample with all complete data included 9218 participants. Overall, 52.2% of the sample consisted of women and the mean age was 35.8 (SD: 14.0) years. The percentages reporting ≥10 min/week walking and cycling for transport were 75.2% and 9.7%. The percentages reporting ≥10 min/week walking for transport ranged between 59.9% (Venezuela) and 83.5% (Costa Rica). While cycling for transport ranged between 2.5% (Venezuela) and 15.9% (Costa Rica). The levels of ≥10 min/week cycling for transport in the last seven days were much lower in contrast to ≥10 min/week walking for transport.

The perceived neighborhood built environment varied greatly across countries. Land use mix-diversity was the highest in Colombia (mean: 3.1; SD: 0.7), and the lowest in Venezuela (mean: 2.4; SD: 0.8) assessed with a 5-point scale. The differences in means of the other environmental variables across the countries were relatively small, about 0.6, in the variables assessed with a 4-point scale. The overall mean scores of proximity of public open spaces (mean: 3.3; SD: 1.1) and of shopping centers (mean: 4.0; SD: 1.3) (5-points scales) indicated greater perceived proximity of public open spaces than to shopping centers. The mean scores of the items of street connectivity and safety from traffic were similar across all countries (differences ≤ 0.4) (Table 3).

### 3.2. Perceived Neighborhood Built Environmental and Walking for Transport

Estimated associations of perceived neighborhood built environment subscales with walking for transport are shown in Table 4. In overall, perceived land use mix-access (OR: 1.32; 95%CI: 1.16, 1.50) and the existence of many alternative routes in the neighbourhood (OR: 1.09; 95%CI: 1.01, 1.17) were associated with higher odds of reporting any walking for transport (defined as ≥10 min/week). Perceived slow speed of traffic (OR: 1.88; 95%CI: 1.82, 1.93) and few drivers exceeding the speed limits (OR: 1.92; 95%CI: 1.86, 1.98) were also related to higher odds of reporting any walking for transport.

There were different associations among countries between walking for transport and perceived neighborhood built environment subscales. Argentina was the country with the strongest associations between perceived aspects of the neighborhood built environment (land use mix-diversity, land use mix-access, aesthetics, safety from crime, streets in neighbourhood do not have many cul-de-sacs, the existence of many alternative routes in the neighbourhood, much traffic, and most drivers exceed the posted speed limits) and walking for transport (Table 4).

### 3.3. Perceived Neighborhood Built Environmental and Cycling for Transport

Estimated associations of perceived neighborhood built environment subscales with cycling for transport are presented in Table 5. In the overall analyses, the odds of reporting any cycling for transport (defined as ≥10 min/week) were higher in participants perceiving more walking/cycling facilities (OR: 1.87; 95%CI: 1.76, 1.99), and better aesthetics (OR: 1.22; 95%CI: 1.09, 1.38).

Distinct associations by country were observed between perceived neighborhood built environment characteristics and cycling for transport. Brazil was the country with the strongest associations between perceived aspects of the neighborhood built environment (land use mix-diversity, and proximity of public open space, and shopping centers) and cycling for transport (Table 5).

## 4. Discussion

The present study aimed to investigate whether different perceived built environment characteristics are associated with walking and cycling for transportation. Our main findings were that land use mix-diversity and mix-access, the presence of different alternative routes to access the destination, lower speed limit in the roads, as well as the majority of the drivers respecting speed limit, were associated with higher walking for transportation. The presence of walking or cycling facilities and a higher aesthetics were associated with higher cycling for transportation. We also highlight some regional specific associations land use mix-diversity (Argentina, Brazil, and Colombia), aesthetics (Argentina, Brazil, and Peru), safety from crime (Argentina and Chile - inverse), proximity to public open spaces (Chile), few dead-end streets (Argentina) and low traffic along the nearby streets (Argentina and Venezuela) were associated with higher odds of walking for transport in specific countries. Similarly, land use mix-diversity (Brazil), land use mix-access (Peru), proximity to public open spaces (Brazil and Costa Rica), few dead-end streets (Colombia), the distance between intersections in the neighbourhood (Ecuador), different alternative routes to access the destination (Costa Rica) and the majority of the drivers respecting speed limit (Costa Rica) were associated with higher odds of cycling for transport in specific countries.

Our findings regarding walking for transport are in line with previous findings from economic developed countries [12,13]. In this sense, we found that factors related to walkability as connectivity as well as related to traffic safety were the most associated with higher walking for transport. These findings highlight the role of planning compact cities aiming to shift the transportation mode towards higher active transportation through increasing the land use mix-access and connectivity [38,39].

In addition, our findings support the important role of traffic safety on walking for transportation. Therefore, measures for traffic calming and safety as lowering the speed limit of the roads can increase physical activity during transportation as well as reduce road injuries [40] and consequently, impact secondary health outcomes, producing a disability-adjusted life-years gain [38].

The findings also pointed out the role of aesthetics in transportation through cycling. Even the majority of evidence found that aesthetics is important most for leisure-time activities as walking during leisure-time [12,41,42], some previous studies also found an association between aesthetics and physical activity during transport [12,13,43], therefore, a pleasant environment seems to be determinant for the choice of cycling for transportation. Another finding was related to the association of cycling facilities with the adoption of cycling for transportation, which highlightes the need for urban infrastructure supporting cyclists, as the build of bike paths as well as the expansion of integrated transportation systems with infrastructure for cyclists, including bike-sharing and parking [44,45].

Our findings showed that there were more perceived environmental correlates of walking than cycling for transportation across countries. Country-specific associations can guide specific policies for each country included in the present study. For example, different correlates related to connectivity, land use, safety from crime, aesthetics and safety from traffic were associated with walking for transportation in Argentina, while only aesthetics and land use mix-diversity were associated with walking for transportation in Brazil. Similarly, Costa Rica presented the highest number of correlates of cycling for transportation, including the distance of public open spaces, the number of alternative routes and few drivers exceeding the speed limit, while other countries presented more specific unique determinants as land usemix-access for Peru and other did not present correlates as Argentina, Chile, and Venezuela.

Our results shows positive association between walking for transport and land use mix-diversity in Argentina, Brazil and Colombia; land use mix-access was positively associated in overall, Argentina, Colombia, and Venezuela. We found an unequal association between the neighborhood built environment characteristics (e.g., aesthetics [Argentina, Brazil, and Peru], safety from crime [Argentina, and Chile], distance to public open spaces [and Chile]) and walking for transportation by country. The documentary analysis of the ten Latin American cities show several programs in Latin America that positively impact active transportation [44]. Several cities have implemented policies and programs aimed promoting walking and cycling for transport. Some of the most relevant and commonly implemented programs are the Ciclovia Recreativa Programs, and the implementation of Bus Rapid Transit systems (BRT) projects [44,46]. In Latin America, BRT systems have been implemented in Brazil, Ecuador, Peru, Mexico, and Colombia where they are considered an efficient and cost-effective solution for urban mobility [47]. Although the main objective of BRT is to increase urban mobility and reduce transport-related time, they also have the potential to stimulate the use of walking and cycling for transport and reduce car ownership use, thus promoting prysical activity [48].

Despite the health benefits of these systems implemented in Latin American countries, private car ownership in this region has been increasing steadily [49,50] such as Brazil, Argentina, Chile, and Colombia [51]. Studies show that in high-income countries, increasing car ownership does not necessarily lead to an increase in car-usage [52]. However, lower use of non-motorized modes of transport, the higher social advantage offered by owning a car, unequal public transport systems and the trend of increasing car ownership is likely to have a negative influence on walking and cycling for transport [53,54].

In our results, the odds of reporting cycling for transport was higher in respondents living in neighborhoods perceived to be more aesthetically pleasing and better walking/cycling facilities. It may be that aesthetically pleasing environments and green spaces can act as motivators for engaging in or spending more time in active transportation [55]. The way aesthetics are associated with active transportation has an important implication [13]. The aesthetics may play a different role in deciding whether or not to engage in different types of physical activity. While aesthetics may not be a relevant environmental feature for walking, it may play a more relevant role for involvement in moderate-to-vigorous physical activity [56]. Aesthetics ratings, like safety ratings, were low across all coutries, and this may be an area for improvement with less cost implications than other structural changes.

Some limitations should be considered in the interpretation of our study. These data do not include car ownership that is likely to have a negative influence on walking and cycling for transport [53,54]. We used self-reported measures of the built environment (perceived) and physical activity, which can lead to recall bias. Also, the cross-sectional design does not allow a causality interpretation, even the reverse-causality is not probable after the adjustment for sociodemographic factors as socioeconomic status. The use of self-report measures of both neighborhood environments and physical activity should be considered in the interpretation of the findings since there is evidence that items from the IPAQ—long (the measure of physical activity used in this study) may be interpreted differently across different cultures and contexts [57]. Also, the NEWS scale has been found to assess density and access to services more accurately in low-to-medium density urban environments [17]. Considering these measurement issues, some of the between-country differences in associations observed in this study might have been due to differences in the interpretation of the survey items. Furthermore, low variability within countries and the small sample sizes in some countries, especially for cycling, may lead to non significant coefficients for variable that affect walking or cycling. This study has several strengths. We presented data on the association between perceived built environment and transportation physical activity (walking and cycling) from eight different Latin-American countries, which enables a regional as well as country-specific vision with more than 9000 participants. By providing a unique Latin American dataset, the present study enabled wider cross-country comparisons and, thus, expanded the existing literature. In addition to identifying neighborhood built environment characteristcs associated with physical activity that might inform public health policies and investments, our findings should be viewed as an opportunity to inform and motivate researchers in Latin America to further examine these relationships. Prospective studies of environmental characteristics and active transportation are needed as well as evidence from intervention studies to better inform policy changes and large-scale environmental interventions.

## 5. Conclusions

This study showed the importance of environmental characteristics for walking and cycling for transport. In general lines, improving perceptions of neighbourhood built environment through changes in the actual neighbourhood built environment could be a target for increasing active transport among inhabitants from Latin America. Programs and policies should consider differences by country. For example, land use mix-diversity was consistently associated with higher walking and cycling for transportation in Brazil, while the respect to the speed limits by drivers was consistently associated with walking and cycling for transportation in Costa Rica. Future studies should investigate the prospective association of environmental characteristics with change in transport physical activity among Latin America countries.

## Figures and Tables

**Table 1:** Summary of environmental scales, NEWS-A.

Scale	Items
Land use mix-diversity	About how long would it take to get from your home to the nearest businesses or facilities listed below if you walked to them? Items: convenience/small grocery store, supermarket, blacksmith, fruit/vegetable market, laundry/dry cleaners, clothing store, post office, library, university/school, other educational centers, book store, fast food restaurant or street food, bakery/coffee shop, bank, non-fast food restaurant, video store, pharmacy/drug store, salon/barber shop, your job or school, public transport stop, park or square, gym or fitness facility
Land use mix-access	Stores are within easy walking distance of my home. It is easy to walk to a transit stop (bus, train) from my home. There are many places to go within easy walking distance of my home. The streets in my neighborhood are hilly, making my neighborhood difficult to walk in (reversed). There are major barriers to walking in my local area that make it hard to get from place to place (for example, freeways, railway lines, rivers) (reversed).
Street connectivity	The streets in my neighborhood do not have many cul-de-sacs (dead-end streets). The distance between intersections in my neighborhood is usually short (100 yards or less; the length of a football field or less). There are many alternative routes for getting from place to place in my neighborhood. (I don’t have to go the same way every time).
Walking/cycling facilities	There are sidewalks on most of the streets in my neighborhood. Sidewalks are separated from the road/traffic in my neighborhood by parked cars. There is a grass/dirt strip that separates the streets from the sidewalks in my neighborhood.
Aesthetics	There are trees along the streets in my neighborhood. There are many interesting things to look at while walking in my neighborhood. There are many attractive natural sights in my neighborhood (such as landscaping, views). There are attractive buildings/homes in my neighborhood.
Safety from traffic	There is so much traffic along nearby streets that it makes it difficult or unpleasant to walk in my neighborhood (reversed). The speed of traffic on most nearby streets is usually slow (50 km/h or less) Most drivers exceed the posted speed limits while driving in my neighborhood (reversed) There are crosswalks and pedestrian signals to help walkers cross busy streets in my neighborhood.
Safety from crime	My neighborhood streets are well lit at night. Walkers and bikers on the streets in my neighborhood can be easily seen by people in their homes. There is a high crime rate in my neighborhood (reversed). The crime rate in my neighborhood makes it unsafe to go on walks during the day (reversed). The crime rate in my neighborhood makes it unsafe to go on walks at night (reversed). The parks, public squares, green areas and recreation areas in my neighborhood are unsafe during the day (reversed).* The parks, public squares, green areas and recreation areas in my neighborhood are unsafe at night (reversed).*

* items not in the NEWS-A scale.

**Table 2:** Demographic characteristics and walking and cycling for transport.

Variables	Overall	Argentina	Brazil	Chile	Colombia	Costa Rica	Ecuador	Peru	Venezuela
Sample size (n)	9218	1266	2000	879	1230	798	800	1113	1132
Age, mean (SD)	35.8 (14.0)	36.8 (13.9)	36.5 (13.8)	36.4 (14.2)	36.9 (14.6)	35.2 (13.9)	34.3 (14.0)	34.2 (13.6)	35.0 (13.8)
Sex (%)									
Men	47.8	45.3	47.1	48.4	49.0	49.4	49.6	47.0	48.8
Women	52.2	54.7	52.9	51.6	51.0	50.6	50.4	53.0	51.2
Socioeconomic level (%)									
Low	52.0	48.7	45.8	46.8	63.3	32.8	49.9	47.9	77.7
Medium	38.4	46.2	45.8	44.1	31.2	53.6	37.1	31.9	16.8
High	9.5	5.1	8.5	9.1	5.4	13.5	13.0	20.2	5.5
Walking for transport ≥ 10 min/week (%)	75.2	69.0	72.6	75.1	79.3	83.5	85.0	85.4	59.9
Cycling for transport ≥ 10 min/week (%)	9.7	10.7	11.3	12.9	9.9	15.9	9.3	6.6	2.5

SD: standard deviation.

**Table 3:** Overall and country perceived-environment scores.

	Overall	Argentina	Brazil	Chile	Colombia	Costa Rica	Ecuador	Peru	Venezuela
Land use mix-diversity (score 1–5)	2.8 (0.8)	2.9 (0.8)	2.6 (0.8)	2.6 (0.6)	3.1 (0.7)	2.8 (0.8)	3.0 (0.6)	2.7 (0.7)	2.4 (0.8)
Land use mix-access (score 1–4)	3.0 (0.4)	3.2 (0.4)	3.0 (0.4)	3.2 (0.4)	2.9 (0.4)	3.2 (0.4)	2.9 (0.4)	3.0 (0.4)	3.0 (0.4)
Walking/cycling facilities (score 1–4)	2.8 (0.6)	2.9 (0.5)	2.7 (0.6)	3.2 (0.6)	2.7 (0.5)	2.8 (0.8)	2.6 (0.4)	2.6 (0.7)	2.8 (0.6)
Aesthetics (score 1–4)	2.6 (0.7)	2.6 (0.7)	2.5 (0.7)	2.9 (0.8)	2.7 (0.6)	2.6 (0.7)	2.4 (0.6)	2.3 (0.7)	2.6 (0.7)
Safety from crime (score 1–4)	2.5 (0.6)	2.4 (0.5)	2.4 (0.6)	2.8 (0.6)	2.6 (0.5)	2.6 (0.6)	2.6 (0.5)	2.6 (0.5)	2.2 (0.6)
Proximity to public open spaces (score 1–5)	3.3 (1.1)	3.0 (1.0)	3.6 (1.0)	2.6 (0.8)	3.3 (1.0)	2.7 (0.9)	3.4 (0.9)	3.6 (1.0)	3.8 (1.1)
Proximity to shopping centres **^(1)^**	4.0 (1.3)	3.1 (1.5)	4.5 (0.9)	4.0 (1.2)	4.0 (1.2)	3.5 (1.4)	4.3 (1.0)	4.1 (1.2)	3.9 (1.4)
Street connectivity items **^(2)^**									
The streets in my neighborhood do not have many cul-de-sacs (dead-end streets).	2.5 (0.9)	2.6 (1.0)	2.7 (0.9)	2.7 (1.1)	2.5 (0.8)	2.4 (0.9)	2.3 (0.8)	2.3 (0.8)	2.5 (0.9)
The distance between intersections in my neighborhood is usually short (100 yards or less; the length of a football field or less).	2.8 (0.8)	3.0 (0.8)	2.7 (0.8)	3.0 (0.9)	2.9 (0.7)	2.9 (0.8)	2.8 (0.7)	2.9 (0.8)	2.7 (0.8)
There are many alternative routes for getting from place to place in my neighborhood. (I don’t have to go the same way every time.)	3.0 (0.8)	3.1 (0.8)	3.0 (0.8)	3.2 (0.8)	3.0 (0.7)	3.1 (0.8)	3.0 (0.7)	3.0 (0.7)	3.0 (0.7)
Safety from traffic items **^(2)^**									
There is so much traffic along nearby streets that it makes it difficult or unpleasant to walk in my neighbourhood. (reversed)	2.4 (0.9)	2.3 (0.9)	2.3 (0.9)	2.4 (0.9)	2.5 (0.8)	2.3 (1.0)	2.5 (0.8)	2.6 (0.8)	2.4 (0.8)
The speed of traffic on most nearby streets is usually slow (50 km/h or less).	2.6 (0.8)	2.4 (0.8)	2.7 (0.8)	2.6 (0.9)	2.6 (0.7)	2.5 (0.9)	2.6 (0.7)	2.5 (0.7)	2.5 (0.8)
Most drivers exceed the posted speed limits while driving in my neighbourhood. (reversed)	2.3 (0.8)	2.2 (0.8)	2.1 (0.8)	2.3 (0.9)	2.4 (0.8)	2.2 (0.9)	2.3 (0.8)	2.4 (0.8)	2.4 (0.8)
There are crosswalks and pedestrian signals to help walkers cross busy streets in my neighbourhood.	2.4 (0.9)	2.4 (0.9)	2.6 (0.9)	2.8 (0.9)	2.3 (0.8)	2.3 (0.9)	2.4 (0.8)	2.2 (0.8)	2.1 (0.9)

The “street connectivity” and “safety from traffic” items were analyzed individually due to low internal consistency; Results presented as mean (standard deviation). **^(1)^** 5-point scale: 5 min (1), 6–10 min (2), 11–20 min (3), 20–30 min (4), 30+ min (5); **^(2)^** 4-point scale: strongly disagree (1), disagree (2), agree (3), strongly agree (4).

**Table 4:** Multilevel logistic regression models (OR (95%CI)) for walking for transport (0: <10 min/week, 1: ≥10 min/week) by country.

Independent Variables	Overall		Argentina		Brazil		Chile		Colombia		Costa Rica		Ecuador		Peru		Venezuela	
	OR (95%CI)	*p*	OR (95%CI)	*p*	OR (95%CI)	*p*	OR (95%CI)	*p*	OR (95%CI)	*p*	OR (95%CI)	*p*	OR (95%CI)	*p*	OR (95%CI)	*p*	OR (95%CI)	*p*
Land use mix-diversity (score 1-5) **^(1)^**	1.97 (1.90, 2.04)	0.430	1.62 (1.51, 1.75)	<0.001	1.18 (1.02, 1.36)	0.023	1.17 (0.85, 1.59)	0.337	1.68 (1.54, 1.85)	0.001	1.30 (0.99, 1.72)	0.062	1.22 (0.84, 1.78)	0.302	1.00 (0.74, 1.35)	1.000	0.91 (0.75, 1.10)	0.315
Land use mix-access (score 1-4) **^(1)^**	1.32 (1.16, 1.50)	<0.001	1.49 (1.07, 2.08)	0.018	1.06 (0.81, 1.40)	0.671	1.24 (0.80, 1.91)	0.334	3.07 (1.90, 4.95)	<0.001	1.03 (0.64, 1.65)	0.913	0.66 (0.35, 1.27)	0.213	1.16 (0.68, 1.96)	0.586	1.49 (1.08, 2.05)	0.016
Walking/cycling facilities (score 1-4) **^(1)^**	0.97 (0.88, 1.06)	0.454	0.90 (0.68, 1.21)	0.488	0.83 (0.68, 1.00)	0.054	1.03 (0.77, 1.39)	0.820	1.21 (0.88, 1.68)	0.243	1.15 (0.89, 1.50)	0.294	0.83 (0.51, 1.35)	0.459	0.76 (0.55, 1.04)	0.089	0.98 (0.79, 1.21)	0.830
Aesthetics (score 1-4) **^(1)^**	1.03 (0.95, 1.11)	0.517	1.36 (1.07, 1.74)	0.013	1.84 (1.72, 1.99)	0.037	1.04 (0.81, 1.33)	0.786	1.18 (0.87, 1.59)	0.297	1.07 (0.80, 1.42)	0.646	1.03 (0.72, 1.48)	0.862	1.81 (1.29, 2.55)	0.001	0.97 (0.80, 1.18)	0.766
Safety from crime (score 1-4) **^(1)^**	1.05 (0.95, 1.15)	0.344	1.51 (1.16, 1.96)	0.002	1.22 (1.00, 1.49)	0.055	0.71 (0.52, 0.96)	0.027	0.82 (0.59, 1.13)	0.225	0.88 (0.64, 1.23)	0.458	0.86 (0.57, 1.29)	0.460	0.80 (0.53, 1.21)	0.287	1.15 (0.91, 1.46)	0.249
Proximity to public open spaces (score 1-5) **^(2)^**	1.01 (0.95, 1.06)	0.811	0.88 (0.77, 1.02)	0.081	1.06 (0.95, 1.18)	0.339	1.28 (1.03, 1.59)	0.023	0.98 (0.83, 1.16)	0.834	1.05 (0.82, 1.34)	0.712	0.79 (0.61, 1.03)	0.078	1.14 (0.93, 1.39)	0.207	0.98 (0.86, 1.12)	0.777
Proximity to shopping centers **^(2)^**	1.00 (0.95, 1.04)	0.856	1.00 (0.91, 1.11)	0.955	0.99 (0.88, 1,11)	0.854	1.03 (0.89, 1.20)	0.694	0.97 (0.84, 1.13)	0.715	1.05 (0.90, 1.22)	0.567	0.92 (0.73, 1.17)	0.512	0.97 (0.82, 1.14)	0.671	1.05 (0.94, 1.16)	0.395
**Street connectivity items** **^(3)^**																		
The streets in my neighbourhood do not have many cul-de-sacs (dead-end streets).	0.98 (0.93, 1.04)	0.568	1.85 (1.74, 1.97)	0.016	1.05 (0.93, 1.19)	0.409	1.00 (0.85, 1.18)	0.962	1.08 (0.88, 1.32)	0.450	0.88 (0.70, 1.10)	0.251	0.97 (0.75, 1.27)	0.842	1.14 (0.91, 1.42)	0.245	1.05 (0.90, 1.21)	0.541
The distance between intersections in my neighbourhood is usually short (100 yards or less; the length of a football field or less).	1.01 (0.94, 1.08)	0.805	0.98 (0.82, 1.15)	0.770	0.98 (0.86, 1.12)	0.791	0.95 (0.77, 1.17)	0.634	1.07 (0.84, 1.35)	0.596	1.25 (0.97, 1.62)	0.079	1.29 (0.97, 1.73)	0.082	1.06 (0.83, 1.36)	0.623	1,00 (0.85, 1.18)	0.980
There are many alternative routes for getting from place to place in my neighbourhood. (I don’t have to go the same way every time.)	1.09 (1.01, 1.17)	0.021	1.25 (1.05, 1.47)	0.010	0.95 (0.82, 1.10)	0.485	1.17 (0.94, 1.47)	0.167	1.29 (1.02, 1.63)	0.032	0.96 (0.74, 1.25)	0.775	1.15 (0.84, 1.57)	0.393	0.93 (0.70, 1.25)	0.644	1.15 (0.96, 1.39)	0.132
**Safety from traffic items** **^(3)^**																		
There is so much traffic along nearby streets that it makes it difficult or unpleasant to walk in my neighbourhood (reversed).	0.99 (0.93, 1.06)	0.856	1.76 (1.64, 1.90)	0.002	1.11 (0.97, 1.27)	0.136	0.94 (0.78, 1.14)	0.521	0.87 (0.71, 1.08)	0.205	1.03 (0.82, 1.29)	0.810	0.98 (0.74, 1.30)	0.900	1.10 (0.86, 1.42)	0.438	1.22 (1.02, 1.46)	0.028
The speed of traffic on most nearby streets is usually slow (50 km/h or less).	1.88 (1.82, 1.93)	<0.001	0.93 (0.79, 1.09)	0.367	0.92 (0.81, 1.04)	0.181	0.88 (0.73, 1.06)	0.188	0.88 (0.71, 1.08)	0.238	1.73 (1.57, 1.92)	0.008	1.02 (0.76, 1.36)	0.917	0.81 (0.62, 1.04)	0.103	0.86 (0.73, 1.02)	0.092
Most drivers exceed the posted speed limits while driving in my neighbourhood (reversed).	1.92 (1.86, 1.98)	0.016	1.32 (1.09, 1.60)	0.004	1.79 (1.69, 1.91)	0.001	1.01 (0.82, 1.25)	0.903	0.93 (0.75, 1.17)	0.549	1.73 (1.57, 1.93)	0.011	1.21 (0.92, 1.59)	0.177	1.00 (0.77, 1.31)	0.974	0.82 (0.69, 0.98)	0.026
There are crosswalks and pedestrian signals to help walkers cross busy streets in my neighbourhood.	0.99 (0.93, 1.05)	0.689	1.00 (0.86, 1.17)	0.968	1.01 (0.89, 1.16)	0.857	1.06 (0.88, 1.29)	0.542	1.00 (0.83, 1.22)	0.965	1.10 (0.89, 1.37)	0.360	0.80 (0.61, 1.04)	0.098	1.09 (0.87, 1.38)	0.440	0.96 (0.83, 1.13)	0.646

Overall percentage of correct prediction: null model: 75.2%; full model: 82.8%. OR: odds ratio; CI: confidence interval. Multilevel logistic regression model (country as 2nd level) with walking time (0: <10 min/week, 1: ≥10 min/week) as dependent variable, adjusted for sex, age, and socioeconomic level; **^(1)^** higher scores indicate perception of higher land use mix-diversity, higher land use mix-access, more walking/cycling facilities, better aesthetics, and more safety from crime; **^(2)^** higher scores indicate greater proximity; **^(3)^** 4-point scale: strongly disagree (1), disagree (2), agree (3), strongly agree (4).

**Table 5:** Multilevel logistic regression models (OR (95%CI)) for cycling for transport (0: <10 min/week, 1: ≥10 min/week) by country.

Independent Variables	Overall		Argentina		Brazil		Chile		Colombia		Costa Rica		Ecuador		Peru		Venezuela	
	OR (95%CI)	*p*	OR (95%CI)	*p*	OR (95%CI)	*p*	OR (95%CI)	*p*	OR (95%CI)	*p*	OR (95%CI)	*p*	OR (95%CI)	*p*	OR (95%CI)	*p*	OR (95%CI)	*p*
Land use mix-diversity (score 1-5) **^(1)^**	1.10 (0.99, 1.23)	0.085	0.84 (0.63, 1.11)	0.214	1.42 (1.15, 1.75)	0.001	1.45 (0.95, 2.20)	0.084	0.94 (0.69, 1.27)	0.684	1.04 (0.77, 1.40)	0.787	1.48 (0.90, 2.44)	0.121	0.80 (0.53, 1.21)	0.287	0.85 (0.47, 1.53)	0.593
Land use mix-access (score 1-4) **^(1)^**	1.10 (0.91, 1.34)	0.334	1.57 (1.35, 1.91)	0.019	1.17 (0.78, 1.77)	0.446	0.85 (0.48, 1.51)	0.589	1.07 (0.57, 2.00)	0.828	1.19 (0.69, 2.05)	0.522	1.99 (0.88, 4.53)	0.100	2.30 (1.07, 4.93)	0.032	2.39 (0.84, 6.82)	0.103
Walking/cycling facilities (score 1-4) **^(1)^**	1.87 (1.76, 1.99)	0.036	0.89 (0.59, 1.32)	0.548	0.99 (0.75, 1.31)	0.937	0.78 (0.53, 1.15)	0.211	1.21 (0.78, 1.86)	0.399	0.74 (0.56, 0.98)	0.034	0.75 (0.40, 1.42)	0.382	0.83 (0.54, 1.28)	0.410	0.56 (0.27, 1.17)	0.123
Aesthetics (score 1-4) **^(1)^**	1.22 (1.09, 1.38)	0.001	1.46 (1.04, 2.05)	0.029	1.21 (0.95, 1.53)	0.115	1.43 (1.02, 2.02)	0.041	1.42 (0.95, 2.14)	0.091	1.24 (0.91, 1.69)	0.167	1.48 (1.30, 1.79)	0.004	1.10 (0.70, 1.73)	0.688	1.05 (0.57, 1.95)	0.879
Safety from crime (score 1-4) **^(1)^**	0.95 (0.82, 1.09)	0.433	0.94 (0.63, 1.38)	0.734	1.29 (0.96, 1.73)	0.092	0.85 (0.57, 1.26)	0.407	1.44 (1.28, 1.67)	<0.001	1.03 (0.72, 1.46)	0.881	0.79 (0.47, 1.32)	0.365	0.96 (0.54, 1.70)	0.882	1.23 (0.57, 2.65)	0.601
Proximity to public open spaces (score 1-5) **^(2)^**	1.03 (0.95, 1.11)	0.537	1.02 (0.83, 1.26)	0.814	1.31 (1.11, 1.55)	0.001	0.97 (0.74, 1.28)	0.838	0.96 (0.76, 1.21)	0.719	1.64 (1.48, 1.85)	0.002	1.21 (0.85, 1.73)	0.281	0.85 (0.65, 1.12)	0.247	0.92 (0.60, 1.41)	0.705
Proximity to shopping centers **^(2)^**	1.02 (0.95, 1.09)	0.589	0.90 (0.78, 1.05)	0.182	1.21 (1.00, 1.45)	0.047	1.18 (0.96, 1.45)	0.115	0.99 (0.81, 1.20)	0.910	1.21 (1.03, 1.43)	0.024	0.90 (0.69, 1.18)	0.452	0.95 (0.76, 1.19)	0.682	0.96 (0.69, 1.33)	0.799
**Street connectivity items** **^(3)^**																		
The streets in my neighbourhood do not have many cul-de-sacs (dead-end streets).	0.95 (0.88, 1.03)	0.240	0.91 (0.75, 1.11)	0.357	0.99 (0.82, 1.18)	0.872	0.94 (0.76, 1.16)	0.588	1.77 (1.59, 2.00)	0.032	0.99 (0.78, 1.26)	0.954	0.92 (0.65, 1.30)	0.635	1.15 (0.86, 1.54)	0.336	0.99 (0.62, 1.58)	0.971
The distance between intersections in my neighbourhood is usually short (100 yards or less; the length of a football field or less).	0.92 (0.84, 1.01)	0.078	0.95 (0.75, 1.21)	0.691	0.88 (0.72, 1.06)	0.172	1.07 (0.81, 1.40)	0.636	0.96 (0.70, 1.32)	0.795	0.87 (0.67, 1.14)	0.316	0.58 (0.40, 0.85)	0.005	1.32 (0.91, 1.91)	0.145	0.73 (0.43, 1.21)	0.224
There are many alternative routes for getting from place to place in my neighbourhood (I don’t have to go the same way every time).	1.07 (0.96, 1.19)	0.224	1.06 (0.83, 1.37)	0.637	1.00 (0.81, 1.24)	0.976	1.12 (0.81, 1.55)	0.499	0.90 (0.65, 1.25)	0.526	1.62 (1.19, 2.20)	0.002	0.82 (0.55, 1.22)	0.327	0.78 (0.51, 1.20)	0.267	1.23 (0.69, 2.21)	0.485
**Safety from traffic items** **^(3)^**																		
There is so much traffic along nearby streets that it makes it difficult or unpleasant to walk in my neighbourhood (reversed).	1.03 (0.94, 1.13)	0.568	1.08 (0.85, 1.38)	0.523	1.03 (0.84, 1.26)	0.766	1.08 (0.85, 1.38)	0.540	0.87 (0.66, 1.14)	0.309	0.98 (0.78, 1.24)	0.893	1.16 (0.81, 1.66)	0.419	1.22 (0.87, 1.71)	0.258	0.93 (0.53, 1.65)	0.809
The speed of traffic on most nearby streets is usually slow (50 km/h or less).	1.01 (0.92, 1.10)	0.904	0.82 (0.65, 1.03)	0.091	0.96 (0.79, 1.15)	0.642	1.10 (0.86, 1.42)	0.438	1.39 (1.06, 1.74)	0.086	1.22 (0.96, 1.56)	0.100	0.77 (0.52, 1.14)	0.189	1.00 (0.70, 1.41)	0.979	0.99 (0.58, 1.68)	0.963
Most drivers exceed the posted speed limits while driving in my neighbourhood (reversed).	1.09 (0.99, 1.20)	0.089	0.99 (0.76, 1.30)	0.962	1.21 (0.98, 1.49)	0.075	0.78 (0.60, 1.03)	0.077	1.18 (0.89, 1.56)	0.259	1.61 (1.24, 2.10)	<0.001	0.93 (0.65, 1.35)	0.708	0.92 (0.64, 1.33)	0.672	1.08 (0.61, 1.91)	0.793
There are crosswalks and pedestrian signals to help walkers cross busy streets in my neighbourhood.	1.00 (0.92, 1.09)	0.965	0.92 (0.74, 1.16)	0.492	1.07 (0.88, 1.30)	0.514	1.00 (0.77, 1.29)	0.997	1.20 (0.93, 1.55)	0.163	0.82 (0.66, 1.02)	0.080	0.94 (0.65, 1.36)	0.743	1.02 (0.75, 1.39)	0.906	0.95 (0.58, 1.56)	0.842

Overall percentage of correct prediction: null model: 90.3%; full model: 94.7%. OR: odds ratio; CI: confidence interval. Multilevel logistic regression model (country as 2nd level) with cycling time (0: <10 min/week, 1: ≥10 min/week) as dependent variable, adjusted for sex, age, and socioeconomic level; **^(1)^** higher scores indicate perception of higher land use mix-diversity, higher land use mix-access, more walking/cycling facilities, better aesthetics, and more safety from crime; **^(2)^** higher scores indicate greater proximity; **^(3)^** 4-point scale: strongly disagree (1), disagree (2), agree (3), strongly agree (4).
